# Unveiling Neuropsychiatric Phenomena: The Impact of Linguistic Precision on Clinical Insight

**DOI:** 10.1192/j.eurpsy.2025.546

**Published:** 2025-08-26

**Authors:** V. H. Santos, F. M. Tehrani, N. Castro, B. Sousa, Z. C. e Sá, T. Carvalhão, S. Fontes

**Affiliations:** 1 Department of Psychiatry and Mental Health, Cova da Beira Local Health Unit; 2 Faculty of Health Sciences, University of Beira Interior, Covilhã, Portugal; 3 Mental Health Services in the Capital Region of Denmark, Child and Adolescent Mental Health Center, Copenhagen, Denmark; 4 Department of Psychiatry and Mental Health, Viseu Dão-Lafões Local Health Unit, Viseu, Portugal

## Abstract

**Introduction:**

Precision of language in neuropsychiatry is vital for the accurate understanding of complex psychopathological phenomena. Many expressions used in psychiatry, especially those of German origin, reflect nuanced descriptions of patient behaviors, cognitive impairments, and emotional states that are not easily captured by modern terms. These linguistic tools provide a window into the intricate dynamics between the mind and body, helping clinicians interpret and navigate the subtleties of neuropsychiatric conditions.

**Objectives:**

This review aims to explore how specific expressions in neuropsychiatry, derived from clinical German terminology, contribute to a deeper understanding of patient experiences and enhance the precision of clinical assessment. By examining these linguistic elements, the paper seeks to illustrate their relevance in diagnosing and treating neuropsychiatric disorders, particularly where conventional language falls short.

**Methods:**

Through a conceptual analysis, this review delves into the historical development and clinical application of several key terms originating in German psychiatry. Terms such as “Gegenhalten,” which describes paradoxical resistance in catatonia, and “Weltschmerz,” a term encapsulating existential despair, are examined within clinical contexts. The review also discusses other terms such as “Mitgehen,” referring to automatic obedience, and “Vorbeireden,” which highlights disorganized speech patterns. The review draws upon classical psychiatric literature and modern clinical observations to demonstrate how these terms inform diagnosis and treatment strategies.

**Results:**

The use of these specific linguistic constructs offers neuropsychiatrists valuable insights into the subjective experiences of patients, often highlighting behaviors and emotional states that would be otherwise overlooked. For example, “Gegenhalten” allows for the differentiation of motor dysfunction in catatonia, while “Weltschmerz” provides a unique framework for understanding a type of depression that transcends typical diagnostic boundaries. Similarly, “Vobeirreden” aids in the recognition of cognitive disorganization, and “Mitgehen” underscores deficits in volitional control. These terms provide clinicians with greater clarity and precision in diagnosis and therapeutic approaches, bridging the gap between patient experiences and clinical evaluation.

**Conclusions:**

This review underscores the importance of language in the accurate interpretation of neuropsychiatric disorders. It demonstrates how these terms enrich the diagnostic process and offer deeper clinical insights into patient behaviors and symptoms. The nuanced language of neuropsychiatry not only enhances understanding but also serves as a tool for more targeted and effective interventions. Ultimately, this approach encourages clinicians to consider the broader impact of linguistic precision in both diagnosis and treatment planning.

**Disclosure of Interest:**

None Declared

